# Effect of the national reimbursement drug list negotiation policy on utilization of negotiated anticancer drugs in Chinese public hospital: a quasi-natural experiment

**DOI:** 10.3389/fpubh.2025.1662346

**Published:** 2025-12-12

**Authors:** Gang Liu, Cui Li, Hezheng Sun, Huan Liu, Xin Zhang, Kexin Wang, Yanping Wang, Qunhong Wu, Linghan Shan

**Affiliations:** 1 Department of Acupuncture, Second Affiliated Hospital, Heilongjiang University of Chinese Medicine, Harbin, China; 2 Department of Social Medicine, School of Health Management, Harbin Medical University, Harbin, China; 3 Department of Discipline Inspection and Supervision Department, Fourth Affiliated Hospital, Harbin Medical University, Harbin, China

**Keywords:** anticancer drug, national reimbursement drug list negotiation, drug utilization, policy evaluation, China

## Abstract

**Background:**

High prices have limited access to targeted anticancer drugs in China. In 2017, 18 such drugs were added to the National Reimbursement Drug List (NRDL) through national price negotiation.

**Method:**

Monthly hospital procurement data (2015–2019) from 887 public hospitals across 30 provinces were analyzed. Drug utilization was measured by expenditures and defined daily doses (DDDs). A difference-in-differences (DID) model and interrupted time series (ITS) analysis were employed to estimate policy effects.

**Results:**

Following policy implementation, total DDDs of negotiated drugs rose 4.03-fold and expenditures 3.07-fold, both exceeding cancer incidence growth. DID analysis confirmed significant increases for Western medicines (+479,100 DDDs; *p* < 0.001). ITS showed sustained upward trends, especially for Trastuzumab, Bevacizumab, and Abiraterone, while traditional Chinese medicines declined. Bevacizumab demonstrated improved affordability, with higher DDDs but lower expenditures.

**Conclusion:**

The NRDL negotiation substantially improved access to targeted anticancer therapies in public hospitals while containing cost growth. These findings highlight the effectiveness of centralized price negotiation in expanding coverage and equity of high-cost cancer treatments in resource-constrained settings.

## Introduction

In 2019, an estimated 23.6 million new cancer cases and nearly 10 million cancer-related deaths occurred globally, accounting for approximately 250 million disability-adjusted life years (DALYs) (1). The productivity losses, deteriorating health outcomes, and escalating healthcare expenditures associated with cancer have drawn widespread global concern (2–4). In response, targeted therapies have emerged as a cornerstone of oncologic management over the past two decades, owing to their potential to enhance survival outcomes and quality of life for cancer patients (5–7). However, the prohibitive cost associated with these innovative therapeutic drugs constitutes a formidable barrier to patient accessibility, particularly within low- and middle-income countries (8, 9).

In 2020, China accounted for 24% of global newly diagnosed cancer cases and 30% of cancer-related deaths worldwide, with age-standardized incidence (204.8/100,000) and mortality rates (129.4/100,000) both significantly exceeding the global average (10). Despite the escalating cancer burden, targeted anticancer drugs in China were not only more expensive than those in numerous other nations (11), but also frequently unaffordable relative to patients’ average income (12). Per capita anticancer drug spending in China stood at merely USD 4.50, representing 2.6% of the corresponding US spending level (13). This mismatch between the rapidly rising demand for cancer treatment and the limited affordability of effective targeted therapies created a pressing policy problem: many clinically effective drugs existed but were inaccessible to most patients. To address this gap, the Chinese government launched the National Reimbursement Drug List Negotiation (NRDLN) policy in 2017, aiming to reduce prices through centralized negotiation and improve patient access via inclusion in the national insurance system (14).

Many countries have adopted centralized price negotiation to reduce drug costs and improve access. For example, New Zealand uses competitive tendering and negotiations for both patented and generic drugs (15), while Australia’s Pharmaceutical Benefits Pricing Authority negotiates drug prices under the Pharmaceutical Benefits Scheme (16), in the United Kingdom, the National Institute for Health and Care Excellence (NICE) incorporates value-based assessments into negotiations to ensure cost-effectiveness (17), and in Germany, the Federal Joint Committee (G-BA) lists drugs in the statutory insurance formulary after negotiation (18). Collectively, these experiences demonstrate that centralized negotiation can enhance affordability and equity. China’s NRDLN policy represents a similar approach tailored to its national context, aiming to reduce high prices and expand access to anticancer drugs.

The effects of centralized drug negotiation and insurance inclusion remain contentious. Multiple studies have documented that price negotiations substantially reduce patient out-of-pocket expenditures, enhance medication utilization, and decrease domestic drug prices (18–21). Nevertheless, concerns persist that hospitals operating under budget caps or drug expenditure quotas may restrict access to expensive anticancer drugs. Consequently, some patients may be compelled to procure these drugs through retail channels without reimbursement (21, 22).

Despite the growing implementation of centralized price negotiation mechanisms, existing research in China remains predominantly concentrated on individual hospitals or single provinces, offering limited insights into the system-level implications of the NRDLN policy (18–20, 22–24). To date, no nationwide evaluation has systematically assessed how this policy influenced real-world utilization of anticancer drugs within the public hospital sector. This study bridges this research gap by leveraging national-level procurement data and applying quasi-experimental methods to quantify the policy’s impact. Specifically, we employed DID and ITS analyses to estimate changes in drug expenditures and DDDs before and after policy implementation, and examined heterogeneity across therapeutic categories and administration routes.

## Methods

### Data sources

Procurement data were obtained from the CMEI Network, one of the most extensive and authoritative pharmaceutical information platforms in China, which covers more than 1,500 member hospitals across 31 provinces, municipalities, or autonomous regions, primarily consisting of secondary and tertiary public hospitals. These facilities include approximately 55% of all tertiary hospitals nationwide (25), which are the principal providers of oncology diagnosis and treatment. In China, specialized cancer treatment services are restricted to secondary and tertiary hospitals (26). Consequently, despite the underrepresentation of primary and private hospitals, the data remain highly representative of the principal settings for cancer care delivery. A potential selection bias should be acknowledged, as patients treated in lower-level hospitals may not have been adequately represented. Consequently, the generalizability of findings may be primarily confined to the public tertiary system rather than the entire healthcare system. The credibility of this dataset is substantiated by its widespread adoption in peer-reviewed publications within leading international journals (19, 27, 28). For this study, we utilized monthly averages of drug expenditure and DDDs for each medication from January 2015 to December 2019, forming a continuous time series dataset.

### Outcome variables

Two primary outcome measures were analyzed to assess drug utilization:

Defined Daily Doses (DDDs): As recommended by the World Health Organization (WHO), DDDs constitutes a standardized unit of measurement for drug utilization (29), independent of pricing, packaging, or dosage variations. This metric thereby provides an objective reflection of clinical prescription patterns and therapeutic trends. Higher DDDs indicate increased medication consumption within the studied population.

Expenditure: Monthly drug expenditures (in Chinese Yuan, CNY) were recorded at the hospital procurement level and were not adjusted for inflation to reflect real-world purchasing behavior during the policy intervention. The study period (2015–2019) was of limited duration, during which national Consumer Price Index (CPI) fluctuations remained marginal. Consequently, the utilization of nominal values is unlikely to materially bias the interpretation of expenditure trends. Furthermore, given that the substantial growth in DDDs occurred alongside only moderate increases in nominal costs, the conclusion regarding improved affordability remains robust without formal inflation adjustment.

### Data processing and quality control

The procurement data obtained from CMEI undergo multi-stage validation by pharmacists, regulatory experts, and technical staff prior to release. The database aligns all records with a standardized national drug information reference library to ensure uniformity in drug nomenclature, strength specifications, dosage formulations, and packaging sizes.

In this study, we included 887 public hospitals (241 secondary and 646 tertiary) with continuous monthly procurement records for 18 negotiated anticancer drugs and six comparison drugs from January 2015 to December 2019. DDDs were obtained directly from the CMEI database and calculated according to WHO ATC/DDD adult maintenance doses, which inherently accounted for variations in dosage strengths and package sizes. Expenditures were defined as the total monthly procurement cost in nominal CNY; no recalculation of per-DDD pricing was required.

The data exhibited a high level of completeness, with no missing monthly records for the majority of drugs. The sole exception was Chidamide, which presented three non-consecutive months of missing data during the 12-month pre-policy period; these instances were coded as zero procurement quantities and included in the analysis.

**Figure 1:**
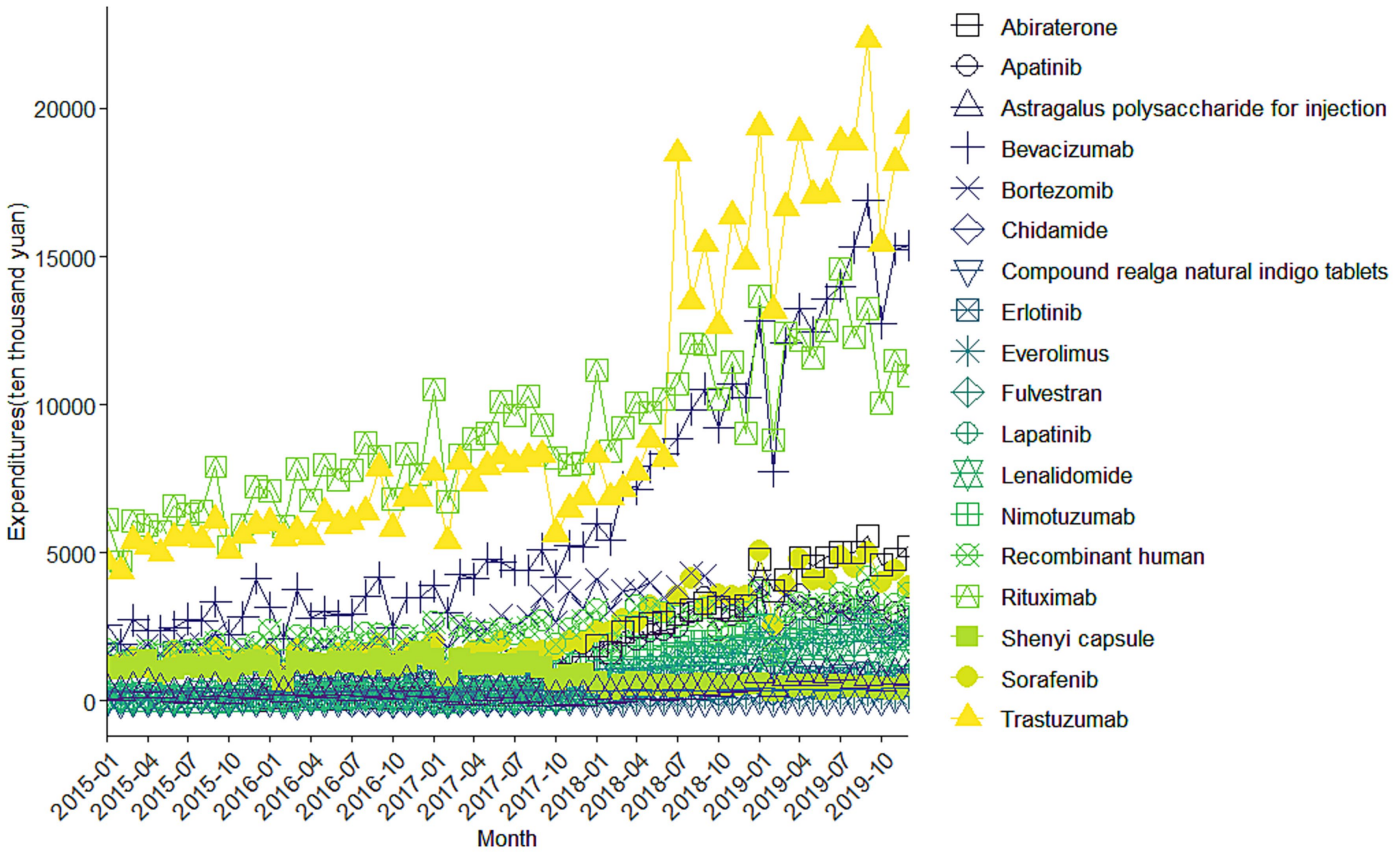
Trends in monthly expenditures of all 18 investigated drugs (January 2015–December 2019). The figures display aggregated procurement data across 887 public hospitals. Because these data represent full national procurement volumes rather than sampled estimates, conventional confidence intervals are not applicable. Statistical inference is provided in the DID and ITS analyses.

**Figure 2:**
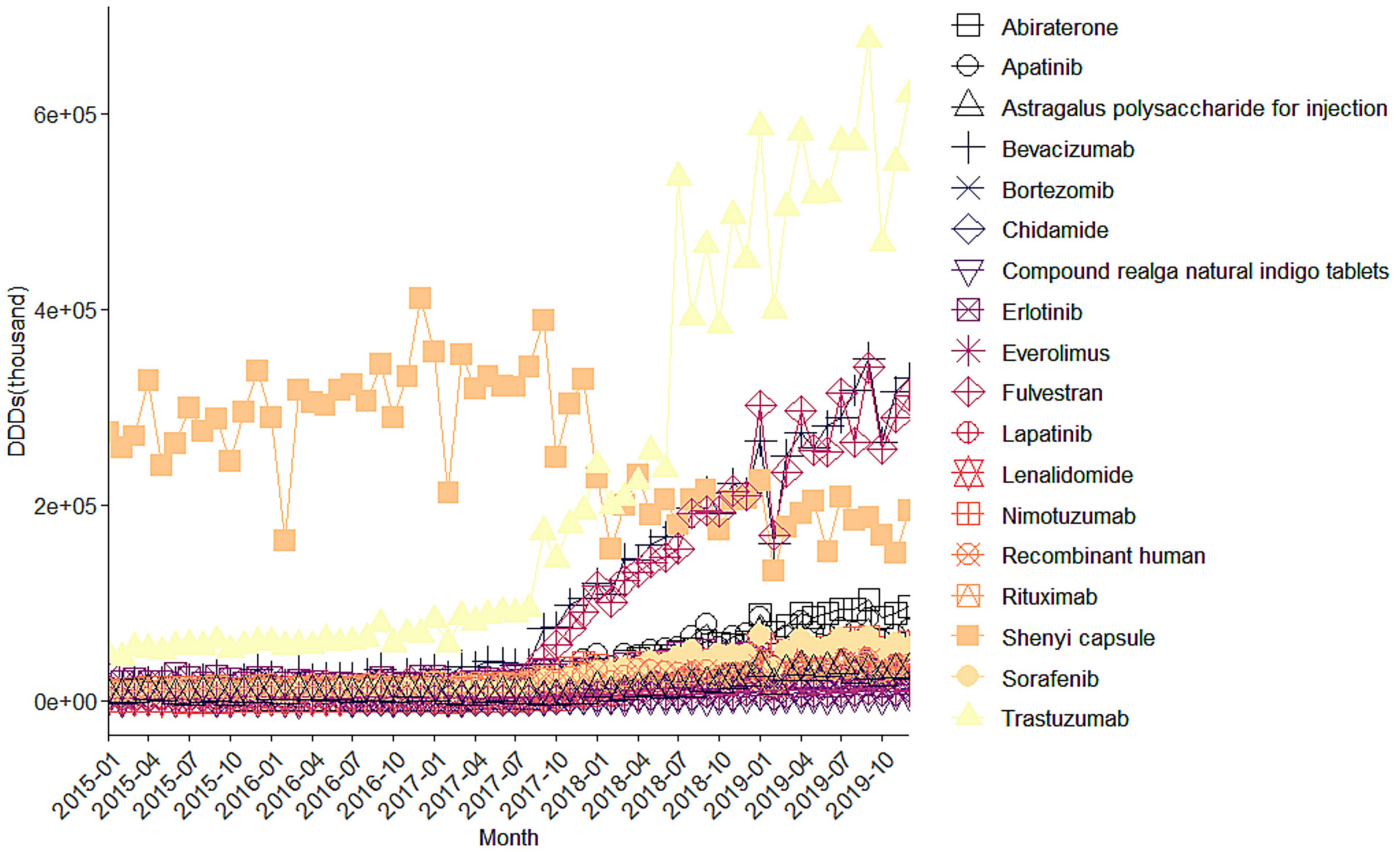
Trends in monthly DDDs of all 18 investigated drugs (January 2015–December 2019). The figures display aggregated procurement data across 887 public hospitals. Because these data represent full national procurement volumes rather than sampled estimates, conventional confidence intervals are not applicable. Statistical inference is provided in the DID and ITS analyses.

As the data represent aggregated procurement volumes across 887 hospitals, as opposed to sampled estimates, descriptive figures (Figures 1–2) are presented without confidence intervals. Statistical inference and confidence intervals are derived from DID and ITS regression models.

### Policy timeframe and groups

The NRDLN policy was officially implemented in July 2017 (month 31 relative to the study’s initiation). Accordingly, we defined the following groups: (a) Intervention group: 15 Western anticancer drugs included in the 2017 NRDLN. (b) Comparison group: Six similar-targeted anticancer drugs excluded from the negotiation (e.g., Cetuximab, Sunitinib). Furthermore, we performed pairwise comparisons between specifically matched drug pairs, such as Bevacizumab (negotiated) versus Cetuximab (non-negotiated), and Erlotinib versus Crizotinib (Table 1).

**Table 1:** Characteristics of anticancer drug in the NRDLN policy intervention group and comparison group.

Category	Code	Generic name	Dosage form and strength	Indications	Market approval date on NMPA#
Intervention group	L01XC02^▲^	Rituximab	100 mg/10 mL, 500 mg/50 mL (Inj)	Non-Hodgkin’s lymphoma (NHL)	April 2000
	L01XC03^▲^	Trastuzumab	440 mg/20 mL (Inj)	Breast cancer, and gastric cancer	September 2002
	L01XC07^▲^	Bevacizumab	100 mg/4 mL (Inj)	Colorectal cancer, and Non-small-cell lung cancer (NSCLC)	February 2010
	XL01XC*	Nimotuzumab	50 mg/10 mL (Inj)	Nasopharyngeal carcinoma (NPC)	April 2008
	L01XE03^▲^	Erlotinib	150 mg, 100 mg (Tab)	NSCLC	April 2006
	L01XE05^▲^	Sorafenib	200 mg (Tab)	Renal cell carcinoma, hepatocellular carcinoma (HCC), and thyroid carcinoma	September 2006
	L01XE07^▲^	Apatinib	250 mg, 375 mg, 425 mg (Tab)	Gastric adenocarcinoma	October 2014
	L01XE07^▲^	Lapatinib	250 mg (Tab)	Breast cancer	January 2013
	L01XX32^▲^	Bortezomib	3.5 mg, 1 mg (Inj)	Multiple myeloma (MM), and lymphoma	February 2005
	L02BA03^▲^	Fulvestran	250 mg/5 mL (Inj)	Breast cancer	January 2010
	L02BX03^▲^	Abiraterone	250 mg (Tab)	Prostatic cancer	May 2015
	L04AA18^▲^	Everolimus	5 mg, 2.5 mg (Tab)	Renal cell carcinoma, endocrine neoplasia, and angiomyolipoma (AML)	January 2013
	L04AX04^▲^	Lenalidomide	10 mg, 25 mg (Tab)	MM	January 2013
	XL01XX*	Chidamide	5 mg (Tab)	Peripheral T cell lymphomas (PTCL)	December 2014
	XL01XX*	Recombinant human endostatin	15 mg/2.4 × 105 U/3 mL (Inj)	NSCLC	September 2005
	ZC01^*^	Compound realga natural indigo tablets	270 mg (Tab)	Acute promyelocytic leukemia (APL)	July 2009
	ZC02^*^	Astragalus polysaccharide for injection	250 mg (Inj)	Antineoplastic adjuvant drugs	August 2004
	ZC02^*^	Shenyi capsule	10 mg (Cap)	Antineoplastic adjuvant drugs	April 2003
Comparison group	L01XC06^▲^	Cetuximab	100 mg/20 ml inj	Colorectal cancer	July 2006
	L01XE04^▲^	Sunitinib	12.5 mg cap	Kidney cancer; Stomach cancer; Pancreatic cancer	October 2007
	L01XX24*	Pegaspargase	5 mL:3750 IU inj	Leukemia	January 2009
	L01XE08^▲^	Nilotinib	200 mg cap,150 mg cap	Leukemia	July 2009
	L01XE16^▲^	Crizotinib	200 mg cap	Lung cancer	January 2013
	L01XE17^▲^	Axitinib	5 mg tab,1 mg tab	Kidney cancer	April 2015

▲ATC Code from the WHO Collaborating Center for Drug Statistics Methodology. *Medicine classification and codes for basic medical insurance. #NMPA, National Medical Products Administration of China.

### Statistical analysis

We utilized two complementary quasi-experimental designs to evaluate the policy impact. The results derived from the DID model constitute the principal findings. Findings from the ITS analysis were reported specifically in instances where trends or effects diverged.

### Difference-in-differences model

The DID approach estimated the average treatment effect of the NRDLN policy by comparing the pre-post changes in outcomes between the intervention and comparison groups. The regression specification was as follows:


$$Y\mathrm{it}=\beta 0+\beta 1\times \mathrm{Pt}+\beta 2\times \mathrm{Ti}+\beta 3(Pt^{*}\mathrm{Ti})+\mathrm{uit}$$

where Y_it_ is the outcome of interest, P is a dummy variable for the second time period and T is a dummy variable for the treatment group. The interaction term, P*T, is equivalent to a dummy variable equals 1 for observations in the treatment group, in the second period.

We inspected the parallel trends assumption using pre-policy trajectory plots (monthly averages by group) and a diagnostic pre-trend regression. Consistent with recent guidance that pre-trend tests are low-power and diagnostic rather than definitive, we treat visualization as the primary check of magnitude and practical relevance of trend differences (30, 31). To triangulate identification when group comparability is imperfect, we additionally use ITS analyses, which do not require cross-group parallelism (32, 33).

The DID approach estimated the average treatment effect of the NRDLN policy by comparing pre-post outcome changes between the intervention and comparison groups. Our specification excluded hospital-level covariates as the CMEI procurement data are aggregated and lack detailed hospital characteristics (e.g., size or case mix). To validate identification, we employed pre-policy trajectory plots and a diagnostic regression test to assess the parallel trends assumption. Additionally, we conducted ITS analyses, which offer robustness under imperfect cross-group comparability.

### Interrupted time series analysis

For each drug, we applied segmented regression models to estimate immediate level changes and changes in slope after the policy. The ITS model specification was:


Yt=β0+β1×Timet+β2×Interventioni+β3×PostTimet+εt

Where Time_t_ is a continuous variable indicating the number of months since the beginning of the study period (August 2016), capturing the baseline trend. Second, Intervention is a binary variable equal to 0 before the implementation of the NRDLN policy and 1 afterward; it measures the immediate level change associated with the policy intervention, reflected by the coefficient $\beta_{2}$ . Third, PostTime_t_ indicates the number of months since the policy was implemented and captures any change in the trend of the outcome, with the effect measured by $\beta_{3}$ Together, $\beta_{2}$ and $\beta_{3}$ allow us to evaluate both the short-term impact and the sustained effects of the NRDLN policy on drug utilization in public hospitals.

#### Autocorrelation diagnostics and correction

We assessed first-order autocorrelation in the residuals of each interrupted time series (ITS) model using the Durbin–Watson (DW) statistic. A DW value substantially below 1.5 or above 2.5 was interpreted as indicating potential autocorrelation. For models exhibiting autocorrelation, we re-estimated the coefficients using Newey–West heteroskedasticity and autocorrelation consistent (HAC) standard errors to obtain unbiased inference. This approach corrects for potential bias in standard errors without altering point estimates, thus ensuring robust significance testing.

#### Multiple hypothesis testing correction

For the 18 ITS analyses conducted (one for each medication), we addressed the multiple testing problem by applying the Benjamini–Hochberg (BH) procedure to control the False Discovery Rate (FDR) at 5% (34). This approach was selected over the Bonferroni correction because the study involved multiple, potentially correlated outcomes (medications), for which Bonferroni is known to be overly conservative, reducing statistical power and increasing the risk of Type II error (35, 36). The FDR method controls the expected proportion of false positives among significant results, which is a more appropriate balance between discovery and error control for health policy evaluations with multiple outcomes (37, 38). We employed the p.adjust function in R (method = “BH”) to adjust *p*-values.

### Software

All statistical analyses were conducted using R version 4.1.2. Statistical significance was set at a two-tailed *p*-value of < 0.05.

### Ethical considerations

This study utilized secondary anonymized data devoid of individual-level patient identifiers. Consequently, ethical approval was not required, and no patients or members of the public participated in the research design or execution.

## Results

### Descriptive analysis

#### Trends in drug utilization: overall and by category

Between 2015 and 2019, the utilization of the 18 negotiated anticancer drugs demonstrated a substantial upward trend. Total hospital procurement expenditure rose by 3.07-fold—from CNY 281,202.12 ten thousand to CNY 862,207.87 ten thousand—while total DDDs increased by 4.03-fold, from 532.65 ten thousand to 2145.51 ten thousand. Following the implementation of the NRDLN policy in mid-2017, utilization accelerated markedly, particularly for Western medicines. Among these, injectable drugs exhibited a 2.87-fold increase in expenditures and a 10.02-fold rise in DDDs, whereas oral drugs increased by 3.97-fold and 1.64-fold, respectively. These figures indicate that injectable therapies became the primary drivers of growth, both in financial and volume terms. In contrast, the three traditional Chinese medicines (TCMs) included in the policy showed a downward trend in both spending and usage, suggesting limited responsiveness to the negotiation policy.

When disaggregated by drug class, monoclonal antibodies (e.g., Trastuzumab, Bevacizumab) exhibited the most significant increases, with expenditures rising from CNY 183955.59 ten thousand to CNY 551246.36 ten thousand, and DDDs growing from 115.12 ten thousand to 1092.12 ten thousand. Protein kinase inhibitors and endocrine therapies (e.g., Abiraterone, Fulvestrant) also showed substantial upward trajectories. In contrast, all three TCMs experienced a decline in both metrics, with combined expenditures decreasing from CNY 18649.08 ten thousand to CNY 16838.27 ten thousand, and DDDs dropping from 345.97 ten thousand to 257.92 ten thousand (Table 2). The sharp rise in DDDs for injectables compared to orals suggests not only a preference shift in prescribing patterns but also stronger policy-driven supply chain effects for high-cost, hospital-administered agents.

**Table 2:** Descriptive results of both overall yearly expenditures and DDDs in the drug category and Administration and compared drug for each observed year (2015–2019).

	Expenditure (10 thousand yuan)					DDDs (10 thousand)				
Year	2015	2016	2017	2018	2019	2015	2016	2017	2018	2019
18 drug	281202.12	330210.79	403474.66	626757.92	862207.87	532.65	604.85	800.23	1485.71	2145.51
Drug categories										
15 western medicines	262553.04	309595.64	386554.78	614855.50	845369.60	186.67	224.49	409.95	1226.21	1887.59
L01: Antineoplastic agents	258270.89	301784.74	373431.17	550871.97	737614.67	174.02	202.93	356.39	939.62	1395.33
Monoclonal antibodies	183955.59	216082.01	263050.43	385996.27	551246.36	115.12	131.44	240.52	689.45	1092.12
Protein kinase inhibitors	33718.90	39916.18	48776.27	82523.25	105834.90	38.82	47.31	76.73	178.59	227.93
Protease inhibitor	20945.14	21185.99	32900.94	44356.24	34839.81	7.06	7.21	15.88	32.19	26.87
Other antineoplastic agents	19651.27	24600.57	28703.54	37996.21	45693.60	13.02	16.96	23.27	39.40	48.41
L02: Endocrine therapy	1580.58	4117.37	9217.09	47362.83	82257.55	8.94	16.46	46.53	248.43	433.84
L04: Immunosuppressants	2701.57	3693.54	3906.52	16620.71	25497.38	3.71	5.10	7.03	38.17	58.42
3 traditional Chinese medicines	18649.08	20615.15	16919.88	11902.42	16838.27	345.97	380.37	390.28	259.50	257.92
Administration										
Inject	230288.13	269786.99	331435.02	483803.07	659917.06	151.83	180.93	324.80	962.58	1520.72
Table/capsule	50913.99	60423.80	72039.64	142954.85	202290.81	380.81	423.92	475.42	523.14	624.80
Compared group1										
Bevacizumab	32481.69	37860.51	53310.42	101442.09	161566.36	25.08	29.18	62.65	205.14	335.22
Cetuximab	17470.25	17643.99	21569.58	21198.25	50363.49	3.54	3.55	4.69	6.43	33.51
Compared group2										
Erlotinib	15707.89	12347.35	9990.14	9299.17	9473.11	26.06	22.42	27.96	49.26	52.21
Crizotinib	1715.2	3251.91	4135.88	4245.42	39502.05	0.97	1.83	2.33	5.48	76.37

#### Drug-level monthly trends

Figure 1 displays monthly expenditures for the 18 anticancer drugs. Before July 2017, expenditures remained relatively stable, but after the NRDLN policy implementation, total expenditures for multiple drugs increased sharply. This reflects the combined effect of substantial price reductions negotiated through NRDLN and expanded insurance coverage, which lowered financial barriers and stimulated utilization. The largest increase in expenditure was observed for Trastuzumab (CNY 11446.92 ten thousand), Bevacizumab (CNY 10968.18 ten thousand) and Abiraterone (CNY 5042.84 ten thousand). The steepest average monthly growth rates were recorded for Abiraterone (20.4%), Lenalidomide (17.8%), Fulvestrant (15.1%), and Lapatinib (14.1%).

Figure 2 illustrates the monthly DDDs trends. Similar to expenditures, most drugs experienced rapid growth in DDDs after policy implementation, with the exception of Shenyi capsule. Trastuzumab again showed the highest growth in DDDs (+53.07 ten thousand), followed by Fulvestrant (+29.40 ten thousand) and Bevacizumab (+29.30 ten thousand). On average, the monthly growth rate across all 18 drugs was 6.84%, with Abiraterone (22.1%), Chidamide (17.7%), and Lenalidomide (12.9%) demonstrating the most rapid expansion. These trends are consistent with the expected policy mechanisms—price reductions and reimbursement expansion improved affordability and access, thereby accelerating uptake, particularly for high-cost targeted therapies.

#### Comparison with cancer incidence trends

**Figure 3:**
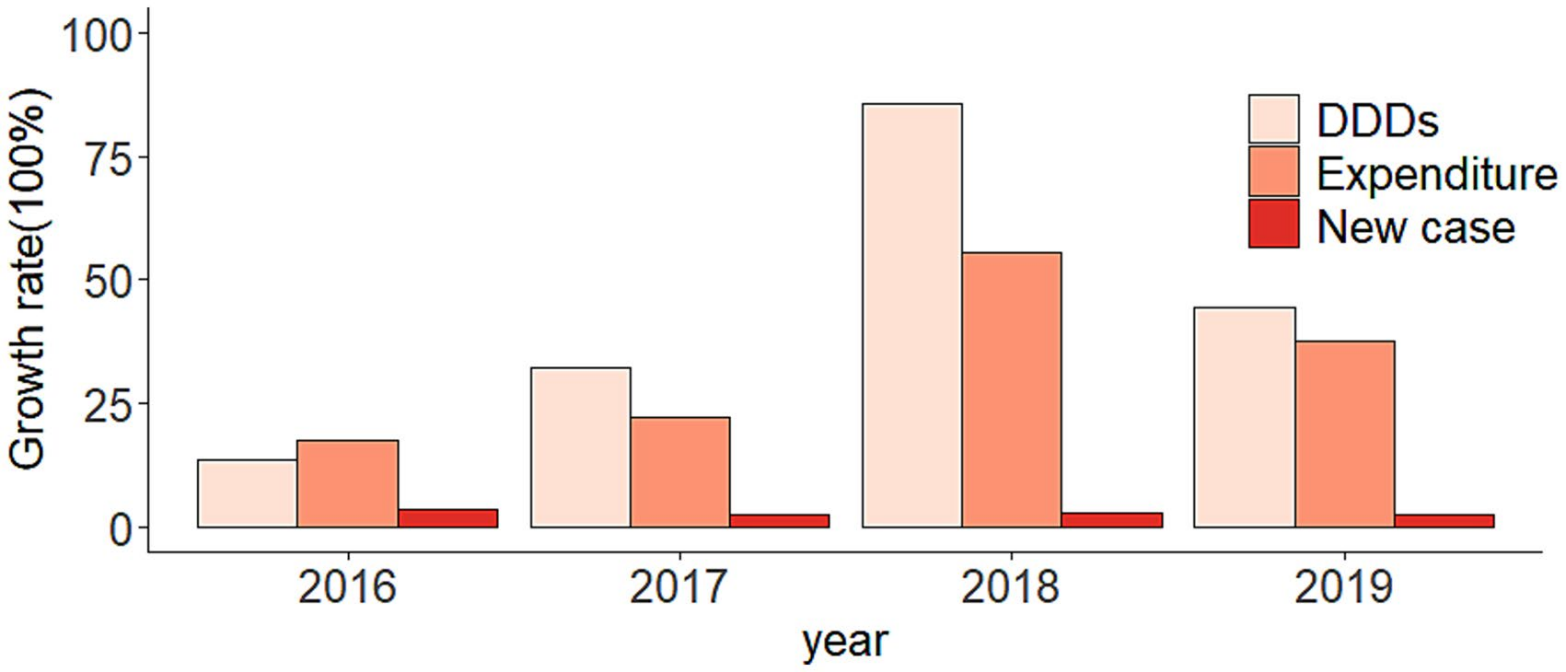
Compare growth rate between cancer new cases and overall anticancer drugs DDDs/expenditure (January 2016–December 2019).

The annual growth rates in expenditure and DDDs for the 18 anticancer drugs exceeded the growth in new cancer cases in China throughout 2016 to 2019 (Figure 3). While the new cancer case rate increased at a relatively stable pace—3.44% in 2016, 2.61% in 2017, 2.88% in 2018, and 2.56% in 2019 (39–42) —the corresponding growth in drug utilization was substantially higher. From 2017 onward, the growth in DDDs outpaced the growth in expenditures, suggesting increasing affordability and broader accessibility.

**Figure 4:**
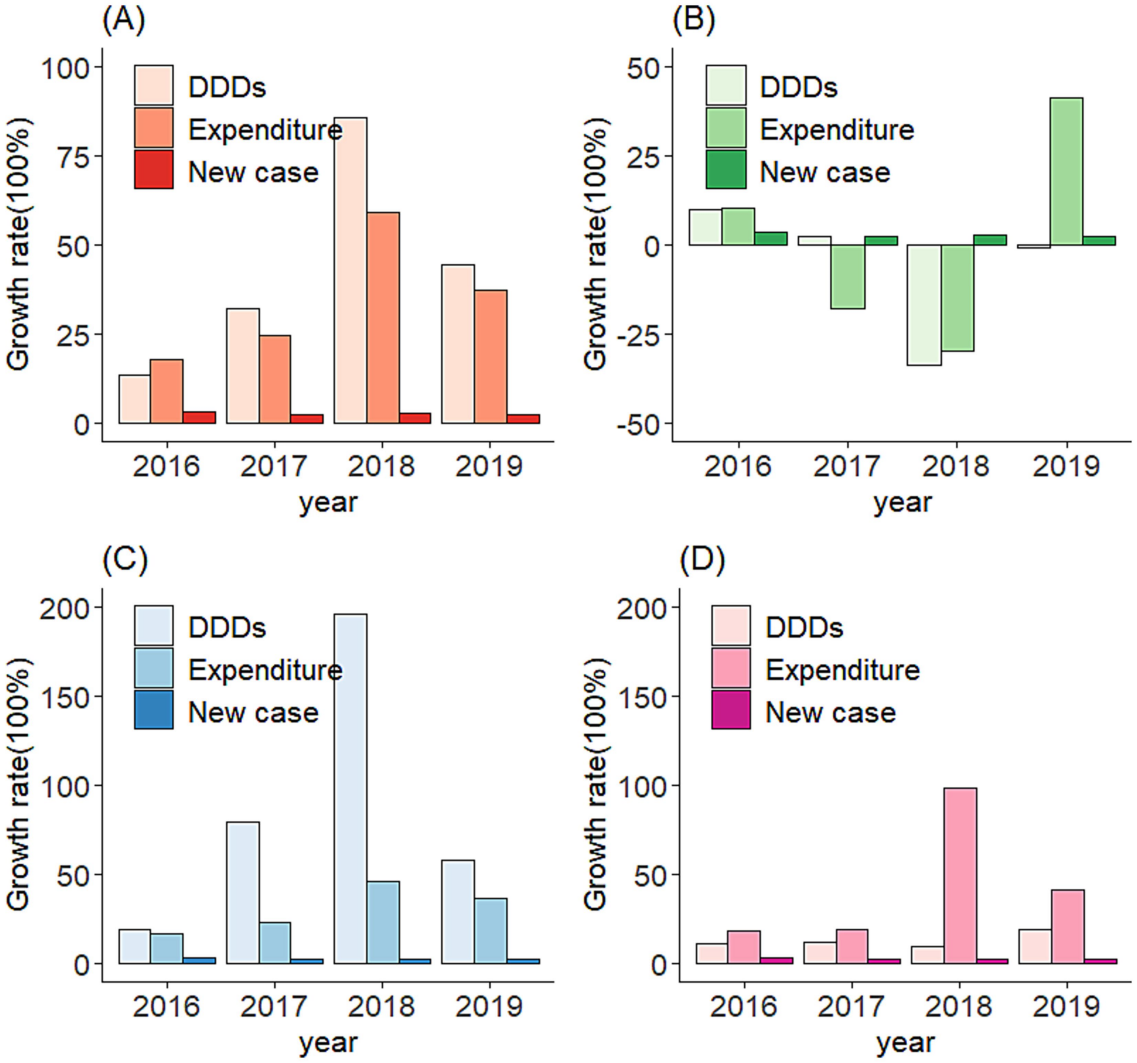
**(A)** Compare growth rate between cancer new case and 15 medicines expenditures/DDDs (from 2016 to 2019). **(B)** Compare growth rate between cancer new case and 3 traditional Chinese medicines expensive/DDDs (from 2016 to 2019). **(C)** Compare growth rate between cancer new case and inject anticancer drugs expenditures/DDDs (from 2016 to 2019). **(D)** Compare growth rate between cancer new case and oral anticancer drugs expenditures/DDDs (from 2016 to 2019).

Further subgroup comparisons (Figure 4) showed that: (a) The 15 Western medicines outperformed the new cancer case growth rate in both DDDs and expenditures. (b) The three traditional Chinese medicines experienced negative growth in both metrics. (c) Both injectable and oral drugs had higher DDD/expenditure growth than cancer incidence, with injectable drugs showing particularly sharp increases post-2017.

Supplementary Tables 1, 2 show that most individual drugs had DDDs growth rates exceeding cancer incidence. However, a few drugs—five in 2016, one in 2017, one in 2018, and two in 2019—had DDDs or expenditure growth rates below the national cancer growth rate.

To enhance interpretability, we converted the increase in DDDs into estimated treatment episodes. Assuming an average treatment intensity of 30 DDDs per month per patient, the observed post-policy increase of approximately 16.1 million DDDs between 2017 and 2019 translates into over 537,000 additional monthly treatment courses. This suggests a substantial expansion in patient-level access to targeted therapies following policy implementation.

#### Difference–in–difference analysis results

**Figure 5:**
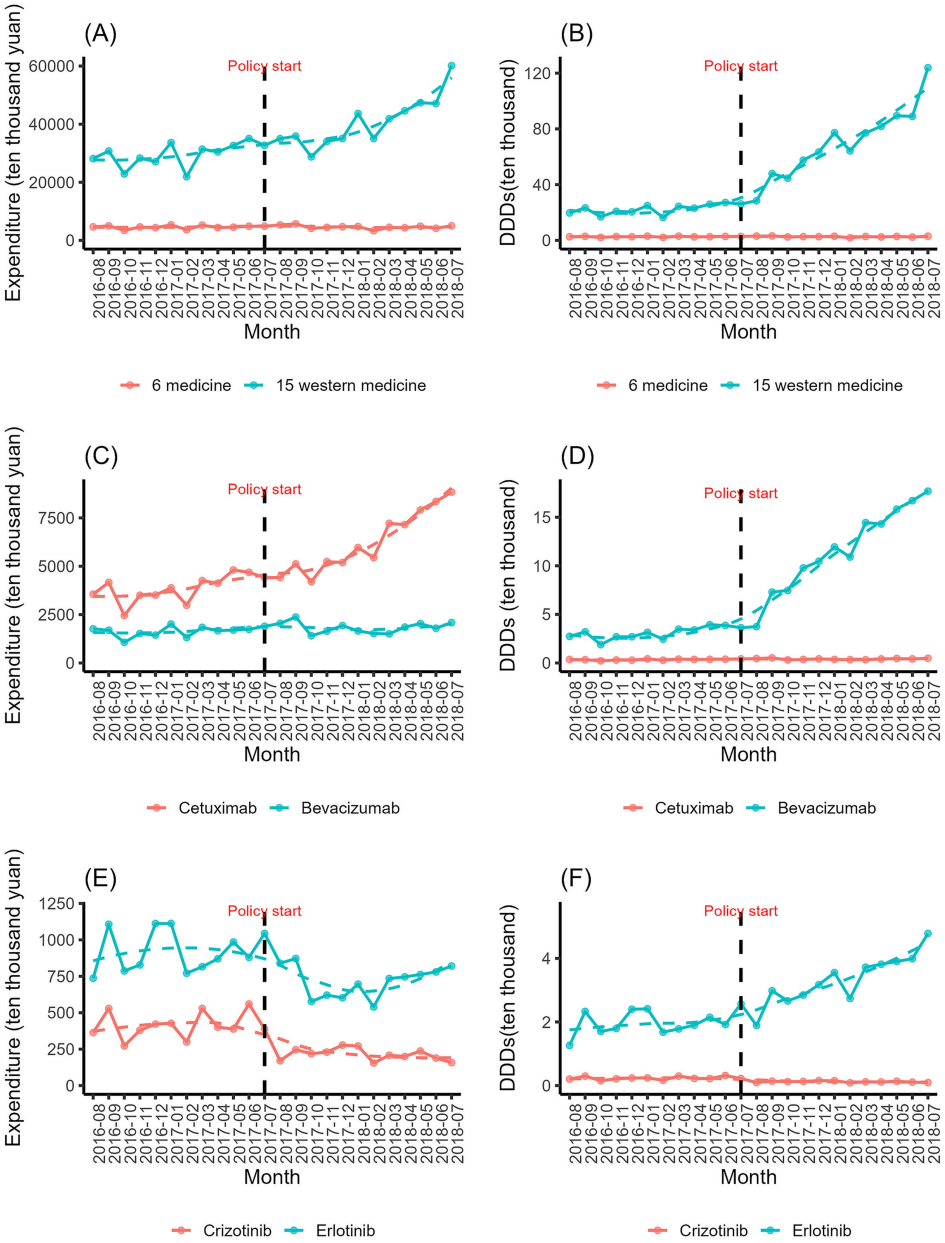
**(A)** Compare pre-policy trajectories between 6 medicine and 15 medicines expenditures (August 2016–July 2017). **(B)** Compare pre-policy trajectories between 6 medicine and 15 medicines DDDs (August 2016–July 2017). **(C)** Compare pre-policy trajectories between Cetuximab and Bevacizumab expenditures (August 2016–July 2017). **(D)** Compare pre-policy trajectories between Cetuximab and Bevacizumab DDDs (August 2016–July 2017). **(E)** Compare pre-policy trajectories between Crizotinib and Erlotinib expenditures (August 2016–July 2017). **(F)** Compare pre-policy trajectories between Crizotinib and Erlotinib DDDs (August 2016–July 2017).

Figure 5 displays monthly pre-intervention trends for the treatment and comparison groups. While pre-trend tests detected statistical differences in slopes, the visual divergence is modest, and ITS estimates—independent of the parallel trends assumption—mirror the DID findings in direction and magnitude, supporting robustness.

The DID analysis confirmed the positive impact of the NRDLN policy on the utilization of anticancer drugs (Table 3). After policy implementation: The 15 negotiated Western anticancer drugs saw a significant increase in expenditure by CNY 11105.20 ten thousand (95% CI: 5598.80–16611.57 ten thousand; *p* < 0.001) and DDDs by 47.91 ten thousand (95% CI: 33.11–62.70 ten thousand; *p* < 0.001). For Bevacizumab, expenditure significantly decreased by CNY 2208 ten thousand (*p* < 0.001), while DDDs increased by 8.56 ten thousand (*p* < 0.001), suggesting improved affordability and access. Erlotinib saw a significant increase in DDDs (+1.46 ten thousand; *p* = 0.008), but its expenditure change was not statistically significant.

**Table 3:** Estimates of effect sizes derived from the difference-in-difference analyses.

Variable	Intervention effect	95% CI	*t*	*p*
15 anticancer western drugs				
Expenditure	11105.20	[5598.80, 16611.57]	4.07	<0.001
DDDs	47.91	[33.11, 62.70]	6.5	<0.001
Bevacizumab				
Expenditure	−2208.30	[−3234.49, −1182.09]	−4.3	<0.001
DDDs	8.56	[6.60, 11.06]	6.9	<0.001
Erlotinib				
Expenditure	−4.91	[−124.02, 114.20]	−0.08	0.934
DDDs	1.46	[0.95, 1.96]	5.84	<0.001

CI, Confidence interval.

#### Interrupted time series analysis results

The ITS analysis further revealed that: No drug exhibited a significant immediate level change (*β*₂) after policy implementation. However, significant changes in post-policy trends (β₃) were observed for 14 drugs in terms of DDDs and 7 drugs in terms of expenditures (Table 4). For example, Abiraterone, Fulvestrant, and Trastuzumab exhibited sustained and statistically significant upward trends in DDDs. Shenyi capsule showed a significant decline in both expenditures and DDDs after 2017. Rituximab, Recombinant human endostatin, and Compound realga natural indigo tablets showed no significant policy effect in either level or trend.

**Table 4:** Results of interrupted time series analysis of 18 negotiated anticancer drugs.

Drug	Drug expenditure (10 thousand yuan)				DDDs (10 thousand)			
	Intercept β0	Baseline trend β1	Level change after MIAN β2	Trend change after MIAN β3	Intercept β0	Baseline trend β1	Level change after MIAN β2	Trend change after MIAN β3
Apatinib	775.180^***^	48.630^*^	−65.160	47.090	1.084^***^	0.067	0.309	0.214^***^
Bevacizumab	3034.890^***^	129.190^*^	−795.170	257.620^**^	2.259^***^	0.131	0.219	1.043^***^
Erlotinib	903.326^***^	1.041	−128.482	−8.743	1.809^***^	0.022	0.147	0.155^**^
Fulvestran	240.150^***^	5.030	8.517	74.466^***^	1.326^**^	0.028	0.619	1.153^***^
Lapatinib	148.196^***^	2.277	−60.781	30.096^***^	0.246^**^	0.004	−0.081	0.102^***^
Lenalidomide	176.754^*^	−3.308	−179.337	110.069^***^	0.133	−0.002	−0.339	0.238^***^
Rituximab	7660.580^***^	137.020	−372.130	−47.980	1.188^***^	0.033	0.218	0.041
Nimotuzumab	1081.436^***^	−4.627	241.746	54.625	1.090^***^	0.020	0.650	0.154^**^
Bortezomib	1586.966^***^	94.929^*^	149.019	−5.587	0.509^*^	0.046	0.368	0.087^*^
Trastuzumab	6294.500^***^	138.700	−1968.800	258.100	5.927	0.246	−0.887	1.963^**^
Sorafenib	1562.260^***^	11.520	−366.910	154.350	0.970^***^	0.007	0.053	0.257^***^
Everolimus	155.586^***^	−2.508	−0.415	7.738	0.314^***^	−0.005	0.068	0.034^**^
Recombinant human endostatin	2028.230^***^	25.320	−72.370	36.030	1.396^***^	0.024	0.166	0.076
Chidamide	−5.779	1.944	2.520	29.036^***^	−0.006	0.002	0.010	0.045^***^
Abiraterone	75.260	11.680	−189.780	241.390^***^	0.061	0.010	−0.223	0.438^***^
Shenyi Capsule	1334.542^***^	−7.976	−77.588	−59.365^*^	32.651^***^	−0.031	2.290	−1.483^*^
Compound realga natural indigo tablets	49.786^**^	−1.299	20.780	3.343	0.1387^**^	−0.003	0.088	0.017
Astragalus polysaccharide for injection	490.004^***^	−20.756^*^	−86.835	36.175^**^	0.939^***^	−0.039	−0.154	0.121^***^

***p < 0.01; **p < 0.01; *p < 0.05.

Out of 36 drug–outcome combinations (18 drugs * 2 outcomes), 41.7% (*n* = 15) exhibited evidence of autocorrelation according to the DW test. After applying Newey–West HAC standard errors, the direction and magnitude of all estimated intervention effects (*β*₃) remained unchanged, and the statistical significance of most coefficients was preserved (Supplementary Tables 3, 4). This indicates that autocorrelation did not materially affect the study conclusions.

Multiple testing correction results:

After applying the FDR correction, all previously statistically significant findings for expenditure and DDDs remained significant at the 5% level. This suggests that our main conclusions are robust to adjustments for multiple comparisons (Supplementary Table 5).

To aid interpretation, key policy-relevant findings are summarized here: (1) Overall anticancer drug utilization rose sharply post-policy, driven mainly by injectables and Western medicines; (2) The cost–volume decoupling observed in drugs like Bevacizumab suggests enhanced affordability; (3) DDDs growth exceeded cancer incidence trends, indicating potential improvements in coverage.

## Discussion

This study demonstrated that the NRDLN policy was associated with a substantial increase in the hospital utilization of negotiated anticancer drugs across China. Both DDDs and expenditures increased markedly, with growth rates exceeding the national rise in new cancer cases. The DID analysis confirmed significant policy effects on overall utilization, while the ITS results revealed sustained—rather than immediate—changes following implementation. Utilization growth was particularly pronounced among Western medicines, especially injectable monoclonal antibodies, while traditional Chinese medicines exhibited declining trends. These findings indicate not only expanded access, but also a structural reallocation of therapeutic resources within the hospital system.

### Expanded utilization with cost containment: evidence of policy effectiveness

This study provides compelling quasi-experimental evidence that the NRDLN policy significantly improved access to negotiated anticancer drugs in Chinese public hospitals. Both drug expenditure and DDDs increased substantially after policy implementation, with DDDs rising more rapidly than expenditures. This divergence signals a reduction in per-unit cost and indicates expanded treatment access within a cost-constrained institutional environment.

The increase in DDDs far outpaced the growth of new cancer cases during the same period, suggesting that the policy not only responded to existing demand but also unlocked previously unmet needs. This reinforces the value of price negotiation as a structural lever to mitigate financial barriers and improve equitable access—particularly in upper-middle-income countries where out-of-pocket drug costs remain high and centralized procurement systems play a dominant role in treatment delivery (43, 44).

### Delayed policy uptake reflects implementation constraints

Despite its effectiveness, the NRDLN policy’s impact was gradual rather than immediate. The absence of significant level changes in ITS analysis, combined with the progressive trend increases, reflects the decentralized and staggered rollout across provinces. In practice, the policy announcement in July 2017 was not uniformly translated into operational changes until several months later in most regions. This underscores a well-recognized gap between policy formulation and administrative execution in large, heterogeneous health systems.

To accelerate translation from policy intent to patient-level benefit, future reforms should prioritize enhanced alignment of national and provincial purchasing schedules, synchronized drug formulary updates, and targeted provider-level guidance. These institutional bottlenecks—rather than policy content itself—likely shaped the observed implementation lag.

### Heterogeneous effects reflect drug characteristics and system incentives

The policy’s effect varied markedly by drug type and administration route. Utilization growth was more prominent among Western medicines than TCMs, and among injectable drugs compared to oral formulations. This divergence can be attributed to differences in evidence strength, prescriber confidence, and clinical pathway integration. Western medicines, particularly monoclonal antibodies and kinase inhibitors, are supported by standardized indications and quantifiable clinical endpoints, making them more compatible with formulary-based procurement and insurance reimbursement systems. For instance, trastuzumab has been established as a guideline-recommended therapy for HER2-positive breast and gastric cancers, where it substantially improves response rates and survival outcomes when combined with chemotherapy (45–47). In contrast, TCMs—often adjunctive in nature—lack consistent clinical metrics and thus are more likely to be deprioritized under performance-based or quota-sensitive purchasing regimes (48). A representative example is Shenyi Capsule, which is mainly used as an adjunct to chemotherapy rather than as a stand-alone anticancer treatment (49); when more effective curative drugs become affordable, hospitals and patients may naturally shift away from such adjuvant options.‌‌

Injectable drugs demonstrated sharper increases in DDDs than oral drugs. This may reflect both clinical appropriateness and systemic incentives: injectable agents are typically administered in hospital-based settings, which facilitates institutional monitoring, quality assurance, and integration into bundled payment schemes. Oral therapies, though potentially more convenient, may face adoption barriers due to lower insurance reimbursement, limited physician control, or weaker procurement incentives.

### Improving access without increasing spending: the bevacizumab case

The case of Bevacizumab illustrates the policy’s dual effect of increasing access while containing costs. Despite a substantial rise in DDDs, total expenditures decreased, reflecting the magnitude of negotiated price reductions. Bevacizumab’s expanded indications—covering multiple high-incidence cancers in China—and strong clinical benefit profile (50–52) likely contributed to its rapid uptake. The volume–price decoupling observed in this case is a hallmark of efficient strategic purchasing and suggests that volume-based reimbursement can be achieved without undermining fiscal sustainability.

Such outcomes also highlight how centralized negotiation mechanisms, when well-targeted, can serve as both financial tools and equity-enhancing instruments. The expanded institutional use of previously unaffordable medicines may also reflect broader shifts in care-seeking behavior toward hospitals offering formulary-based coverage, integrated clinical pathways, and reduced out-of-pocket burden (53).

### Potential unintended consequences

Although the NRDL negotiation has substantially reduced drug prices and expanded access, several unintended consequences warrant attention. Previous studies have highlighted concerns regarding potential inequities and budgetary pressures (48). Consistent with these observations, recent empirical evidence shows that disparities persist across provinces and between urban and rural populations, with patients in less developed regions often facing delayed formulary inclusion and higher out-of-pocket costs (12, 28, 54), while price reductions lowered unit costs, hospital expenditures on oncology drugs sometimes increased due to higher utilization, creating new challenges for institutional budgets and long-term financial sustainability (55–57). Prescribing patterns have also shifted toward innovative targeted therapies, consistent with clinical guideline updates, but this trend raises concerns about rational allocation of limited resources (58, 59). Finally, although direct evidence of “crowding out” of older or non-negotiated drugs is limited, hospital budget constraints may indirectly restrict access to other high-cost therapies, underscoring the need for careful monitoring (59, 60).

### Policy implications and future directions

While the NRDLN policy has delivered measurable improvements, its long-term success depends on institutionalizing supporting mechanisms. A stable, transparent HTA system is critical to inform future drug inclusion, price reassessment, and prioritization (61, 62). Real-world evidence should be systematically integrated into post-listing evaluations, enabling dynamic adjustments to reimbursement terms and fostering adaptive benefit design (63).

In parallel, payment systems must evolve to reward value over volume. Metrics such as the utilization–expenditure ratio and population-level treatment coverage should be embedded in hospital and insurer performance assessments. Importantly, such measures must be supported by data infrastructure capable of capturing longitudinal treatment outcomes and utilization patterns.

Finally, while this study demonstrates strong population-level effects, further research is warranted to explore patient-level impacts—including treatment adherence, clinical outcomes, and financial protection. Linking procurement data with clinical registries could support more granular assessments of the policy’s effectiveness, equity, and efficiency.

### Limitations

This study has several limitations. First, the analysis relied on aggregated hospital procurement data rather than individual-level clinical or prescription records. As such, it was not possible to assess the appropriateness of prescribing or patient-level outcomes. For interpretation of affordability and equity, this means that increases in hospital-level DDDs and expenditures primarily reflect supply-side availability rather than confirmed patient-level access. We could not observe out-of-pocket spending, copayment levels, reimbursement rates, or financial toxicity for different patient groups, nor could we determine whether utilization gains accrued disproportionately to patients with better insurance, higher income, or those living in urban/tertiary settings. Similarly, we were unable to assess treatment adherence, discontinuation, dose intensity, or time-to-treatment—all of which shape realized affordability and distributional equity. Nevertheless, procurement volumes remain a valid proxy for institutional drug utilization and reflect real-world supply-side changes.

Second, the DID analysis did not account for hospital-specific characteristics such as size, clinical specialty, or local economic context. Although a matched comparison group was used, unobserved heterogeneity across facilities may have influenced estimates. In addition, although strict parallel trends were not observed for all outcomes, transparent visualization and design triangulation with ITS align with contemporary recommendations for health policy evaluations facing imperfect comparators. The consistency between DID and ITS reduces concern that modest pre-trend differences fully account for the observed policy effects. Future work could extend to formal sensitivity analyses that quantify the degree of parallel-trend violation required to reverse conclusions (31, 64).

Third, due to the limited availability of non-negotiated but clinically identical anticancer drugs, the comparison group was based on therapeutic similarity rather than strict indication-matching. This may introduce clinical context variation, although multiple sensitivity checks across drug classes yielded consistent results.

Fourth, expenditures were not adjusted for inflation. However, the substantial growth in DDDs and the moderate increase in costs suggest genuine gains in affordability rather than price distortion.

Finally, the use of aggregated procurement data limits our ability to assess treatment appropriateness, patient adherence, clinical outcomes, or financial toxicity. Although procurement volumes serve as a valid proxy for institutional utilization, they cannot capture individual-level experiences or health impacts. Future research should link procurement with claims, registries, or discharge abstracts to quantify patient out-of-pocket payments, catastrophic health expenditure, and uptake by insurance type, socioeconomic status, and urban–rural location (e.g., concentration indices or distribution-sensitive DID), and to evaluate adherence and outcomes alongside utilization.

Future research should consider linking procurement datasets with clinical registries, hospital discharge records, or insurance claims to evaluate real-world effectiveness, equity, and patient financial burden. Integrating such data would provide a more comprehensive understanding of the policy’s impact across the full care continuum.

## Conclusion

This study provides robust evidence that China’s 2017 NRDLN policy significantly increased the utilization of negotiated anticancer drugs in public hospitals. The policy was associated with rapid growth in DDDs and expenditures, particularly for Western medicines and injectable formulations, while overall affordability improved. Importantly, the increase in utilization outpaced the national growth in cancer incidence, suggesting that the policy not only expanded coverage but also addressed previously unmet treatment needs. These findings demonstrate the potential of centralized drug price negotiation to promote equitable and efficient access to high-cost therapies within resource-constrained health systems.

Based on these findings, we propose several policy implications. Future reforms should prioritize the institutionalization of a national HTA framework to guide drug inclusion and pricing decisions, the integration of real-world evidence into post-listing evaluations, and the alignment of hospital payment mechanisms with value-based purchasing principles. In addition, efforts to reduce interprovincial implementation delays and to expand dual-channel access through designated retail pharmacies may further enhance the availability of essential cancer treatments. Collectively, these actions can help ensure that centralized negotiation functions not only as a cost-control tool but also as a mechanism for structural equity in access to innovative therapies.

## Data Availability

The original contributions presented in the study are included in the article/Supplementary material, further inquiries can be directed to the corresponding author/s.

## References

[1] Kocarnik JM Compton K Dean FE Fu W Gaw BL Harvey JD . Cancer incidence, mortality, years of life lost, years lived with disability, and disability-adjusted life years for 29 cancer groups from 2010 to 2019: a systematic analysis for the global burden of disease study 2019. JAMA Oncol. (2022) 8:420–44. doi: 10.1001/jamaoncol.2021.6987, 34967848 PMC8719276

[2] Maurya PK Murali S Jayaseelan V Thulasingam M Pandjatcharam J. Economic burden of Cancer treatment in a region in South India: A cross sectional analytical study. Asian Pac J Cancer Prev. (2021) 22:3755–62. doi: 10.31557/APJCP.2021.22.12.3755, 34967553 PMC9080359

[3] Garaszczuk R Yong JHE Sun Z de Oliveira C. The economic burden of Cancer in Canada from a societal perspective. Curr Oncol. (2022) 29:2735–48. doi: 10.3390/curroncol29040223, 35448197 PMC9025082

[4] Cao M Li H Sun D He S Yan X Yang F . Current cancer burden in China: epidemiology, etiology, and prevention. Cancer Biol Med. (2022) 19:1121–38. doi: 10.20892/j.issn.2095-3941.2022.0231, 36069534 PMC9425189

[5] Niraula S Seruga B Ocana A Shao T Goldstein R Tannock IF . The price we pay for progress: a meta-analysis of harms of newly approved anticancer drugs. J Clin Oncol. (2012) 30:3012–9. doi: 10.1200/JCO.2011.40.3824, 22802313

[6] MoniqueElseviers. Drug utilization research (methods and applications). New York: John Wiley and Sons (2016).

[7] Sledge GW. What is targeted therapy? J Clin Oncol. (2005) 23:1614–5. doi: 10.1200/JCO.2005.01.016, 15755966

[8] Prasad V De Jesús K Mailankody S. The high price of anticancer drugs: origins, implications, barriers, solutions. Nat Rev Clin Oncol. (2017) 14:381–90. doi: 10.1038/nrclinonc.2017.31, 28290490

[9] Fundytus A Sengar M Lombe D Hopman W Jalink M Gyawali B . Access to cancer medicines deemed essential by oncologists in 82 countries: an international, cross-sectional survey. Lancet Oncol. (2021) 22:1367–77. doi: 10.1016/S1470-2045(21)00463-0, 34560006 PMC8476341

[10] Cao W Chen H Yu Y Li N Chen W. Changing profiles of cancer burden worldwide and in China: a secondary analysis of the global cancer statistics 2020. Chin Med J. (2021) 134:783–91. doi: 10.1097/CM9.0000000000001474, 33734139 PMC8104205

[11] Goldstein DA Clark J Tu Y Zhang J Fang F Goldstein R . A global comparison of the cost of patented cancer drugs in relation to global differences in wealth. Oncotarget. (2017) 8:71548–55. doi: 10.18632/oncotarget.17742, 29069727 PMC5641070

[12] Sun Y Zhu Z Zhang J Han P Qi Y Wang X . Impacts of national drug price negotiation on expenditure, volume, and availability of targeted anti-cancer drugs in China: an interrupted time series analysis. Int J Environ Res Public Health. (2022) 19:578. doi: 10.3390/ijerph19084578, 35457445 PMC9025142

[13] The IQVIA Institute. *Global oncology trends 2019*. Available online at: https://www.iqvia.com/insights/the-iqvia-institute/reports-and-publications/reports/global-oncology-trends-2019 (Accessed June 17, 2025).

[14] Ministry of Human Resources and Social Security of the People’s of China. *Ministry of human resources and social security releases results of negotiations on national reimbursement drug list entry 2017*. Available online at: https://www.mohrss.gov.cn/SYrlzyhshbzb/shehuibaozhang/zcwj/201707/t20170718_274153.html (Accessed June 17, 2025).

[15] Main B Csanadi M Ozieranski P. Pricing strategies, executive committee power and negotiation leverage in New Zealand's containment of public spending on pharmaceuticals. Health Econ Policy Law. (2022) 17:348–65. doi: 10.1017/S1744133122000068, 35382921

[16] Karikios DJ Chim L Martin A Nagrial A Howard K Salkeld G . Is it all about price? Why requests for government subsidy of anticancer drugs were rejected in Australia. Intern Med J. (2017) 47:400–7. doi: 10.1111/imj.13350, 27928875

[17] Collier J. The pharmaceutical price regulation scheme. BMJ. (2007) 334:435–6. doi: 10.1136/bmj.39136.464421.BE, 17332539 PMC1808121

[18] Hörn H Nink K McGauran N Wieseler B. Early benefit assessment of new drugs in Germany - results from 2011 to 2012. Health Policy. (2014) 116:147–53. doi: 10.1016/j.healthpol.2013.12.008, 24472328

[19] Huang C Ung COL Wushouer H Bai L Li X Guan X . Trends of negotiated targeted anticancer medicines use in China: an interrupted time series analysis. Int J Health Policy Manag. (2021) 11:1489–95. doi: 10.34172/ijhpm.2021.47, 34273922 PMC9808358

[20] Fang W Xu X Zhu Y Dai H Shang L Li X. Impact of the National Health Insurance Coverage Policy on the utilisation and accessibility of innovative anti-cancer medicines in China: an interrupted time-series study. Front Public Health. (2021) 9:714127. doi: 10.3389/fpubh.2021.714127, 34422752 PMC8377668

[21] Luo Z Gyawali B Han S Shi L Guan X Wagner AK. Can locally developed me-too drugs aid price negotiation? An example of cancer therapies from China. Semin Oncol. (2021) 48:141–4. doi: 10.1053/j.seminoncol.2021.03.001, 33875231

[22] Sun W Tang Y Zhou Y Zhang Q Zhang B Du X. Investigation on the clinical application of 17 national medical insurance negotiation anticancer drugs in Peking union medical college hospital. Med J Peking Union Med Coll Hosp. (2020) 12:958–64. doi: 10.3969/j.issn.1674-9081.2020.00.018

[23] Chen Z Leng J Gao G Liu Y. Evaluation of targeted anticancer agents incorporated into medical insurance policy: taking a tertiary oncology institution in Beijing as an example. Chin Health Econ. (2018) 37:30–4. doi: 10.7664/CHE20181007

[24] Xu Y Zhu J Jiang N Li N Song J. Investigation on the effect of 17 national negotiation anti-cancer drugs included in the medical insurance of Tianjin on cancer patients. Chin Prim Health Care. (2020) 34:9–12. doi: 10.3969/j.issn.1001-568X.2020.09.003

[25] Science and Technology Development Center of Chinese Pharmaceutical Association. *Brief introduction to CMEI (2020)*. Available online at: https://zhengwen.cmei.org.cn/file/service.html (Accessed June 17, 2025).

[26] Cao Z Wang L Ma R Hu Y Bao B Liu X . Access to essential and innovative anti-cancer medicines: a longitudinal study in Nanjing, China. BMC Health Serv Res. 24:802. doi: 10.1186/s12913-024-11285-5, 38992687 PMC11242009

[27] Cai L Tao T Li H Zhang Z Zhang L Li X. Impact of the national drug price negotiation policy on the utilization, cost, and accessibility of anticancer medicines in China: a controlled interrupted time series study. J Glob Health. 12:11016. doi: 10.7189/jogh.12.11016, 36527382 PMC9758701

[28] Gao X Yu M Sun Y Zhang T Li X Zhang L . New evidence of the impact of the National Drug Price Negotiation Policy on the availability, utilization, and cost of anticancer medicines in China: an interrupted time series study. Risk Manag Healthc Policy. (2024) 17:2201. doi: 10.2147/RMHP.S473846, 39309121 PMC11414641

[29] WHO Collaborating Centre for Drug Statistics Methodology. *Guidelines for ATC classification and DDD assignment 2019*. WHO Collaborating Centre for Drug Statistics Methodology (2019).

[30] Ryan AM Kontopantelis E Linden A Burgess JF. Now trending: coping with non-parallel trends in difference-in-differences analysis. Stat Med Res. (2019) 28:3697–711. doi: 10.1177/0962280218814570, 30474484

[31] Roth J Sant’Anna PHC Bilinski A Poe J. What’s trending in difference-in-differences? A synthesis of the recent econometrics literature. J Econom. (2023) 235:2218–44. doi: 10.1016/j.jeconom.2023.03.008

[32] Kontopantelis E Doran T Springate DA Buchan I Reeves D. Regression based quasi-experimental approach when randomisation is not an option: interrupted time series analysis. BMJ. (2015) 350:h2750. doi: 10.1136/bmj.h2750, 26058820 PMC4460815

[33] O'Neill S Kreif N Grieve R Sutton M Sekhon JS. Estimating causal effects: considering three alternatives to difference-in-differences estimation. Health Serv Outcomes Res Methodol. (2016) 16:1–21. doi: 10.1007/s10742-016-0146-8, 27340369 PMC4869762

[34] Benjamini Y Drai D Elmer G Kafkafi N Golani I. Controlling the false discovery rate in behavior genetics research. Behav Brain Res. (2001) 125:279–84. doi: 10.1016/s0166-4328(01)00297-2, 11682119

[35] Vickerstaff V Omar RZ Ambler G. Methods to adjust for multiple comparisons in the analysis and sample size calculation of randomised controlled trials with multiple primary outcomes. BMC Med Res Methodol. (2019) 19:129. doi: 10.1186/s12874-019-0754-4, 31226934 PMC6588937

[36] Narum SR. Beyond bonferroni: less conservative analyses for conservation genetics. Conserv Genet. (2006) 7:783–7. doi: 10.1007/s10592-005-9056-y

[37] Voelkl B. Multiple testing: correcting for alpha error inflation with false discovery rate (FDR) or family-wise error rate? Anim Behav. (2019) 155:173–7. doi: 10.1016/j.anbehav.2019.07.001

[38] Shuken SR McNerney MW. Costs and benefits of popular P-value correction methods in three models of quantitative omic experiments. Anal Chem. (2023) 95:2732–40. doi: 10.1021/acs.analchem.2c03719, 36693222 PMC10653731

[39] Zheng R Sun K Zhang S Zeng H Zou X Chen R . Report of cancer epidemiology in China, 2015. Chin J Oncol. (2019) 41:19–28. doi: 10.3760/cma.j.issn.0253-3766.2019.01.005, 30678413

[40] Zheng R Zhang S Sun K Chen R Wang S Li L . Cancer incidence and mortality in China, 2016. Chin J Oncol. (2023) 45:212–20. doi: 10.1016/j.jncc.2022.02.002

[41] China BaoGao.com. *‌2021 China Cancer early screening market analysis report - research on market competition pattern and future trends*. Available online at: https://www.sohu.com/a/479085956_730526.

[42] Feng RM Zong YN Cao SM Xu RH. Current cancer situation in China: good or bad news from the 2018 global Cancer statistics? Cancer Commun (Lond). (2019) 39:22. doi: 10.1186/s40880-019-0368-6, 31030667 PMC6487510

[43] Moye-Holz D van Dijk JP Reijneveld SA Hogerzeil HV. The impact of Price negotiations on public procurement prices and access to 8 innovative Cancer medicines in a middle-income country: the case of Mexico. Value Health Reg Issues. (2019) 20:129–35. doi: 10.1016/j.vhri.2019.04.006, 31374426

[44] Lauenroth VD Kesselheim AS Sarpatwari A Stern AD. Lessons from the impact of price regulation on the pricing of anticancer drugs in Germany. Health Aff Millwood. (2020) 39:1185–93. doi: 10.1377/hlthaff.2019.01122, 32634355

[45] Marty M Cognetti F Maraninchi D Snyder R Mauriac L Tubiana-Hulin M . Randomized phase II trial of the efficacy and safety of trastuzumab combined with docetaxel in patients with human epidermal growth factor receptor 2-positive metastatic breast cancer administered as first-line treatment: the M77001 study group. J Clin Oncol. (2005) 23:4265–74. doi: 10.1200/JCO.2005.04.173, 15911866

[46] Burstein HJ Ln H Pk M Lambert-Falls R Havlin K Overmoyer B . Trastuzumab and vinorelbine as first-line therapy for HER2-overexpressing metastatic breast cancer: multicenter phase II trial with clinical outcomes, analysis of serum tumor markers as predictive factors, and cardiac surveillance algorithm. J Clin Oncol. (2003) 21:2889–95. doi: 10.1200/JCO.2003.02.018, 12885806

[47] Yamamoto D Iwase S Kitamura K Kitamura K Odagiri H Odagiri H . A phase II study of trastuzumab and capecitabine for patients with HER2-overexpressing metastatic breast cancer: Japan breast Cancer research network (JBCRN) 00 trial. Cancer Chemother Pharmacol. (2008) 61:509–14. doi: 10.1007/s00280-007-0497-5, 17516068

[48] Wang S Long S Wu W. Application of traditional Chinese medicines as personalized therapy in human cancers. Am J Chin Med. (2018) 46:953–70. doi: 10.1142/S0192415X18500507, 29986595

[49] Pan L Zhang T Sun H Liu GA-O. Ginsenoside Rg3 (Shenyi capsule) combined with chemotherapy for digestive system cancer in China: a meta-analysis and systematic review. Evid Based Complement Alternat Med. 2019:2417418. doi: 10.1155/2019/2417418, 31929811 PMC6942834

[50] Cheng AL Qin S Ikeda M Galle PR Ducreux M Kim TY . Updated efficacy and safety data from IMbrave150: Atezolizumab plus bevacizumab vs. sorafenib for unresectable hepatocellular carcinoma. J Hepatol. (2022) 76:862–73. doi: 10.1016/j.jhep.2021.11.030, 34902530

[51] Park S Kim TM Han JY Lee GW Shim BY Lee YG . Phase III, randomized study of Atezolizumab plus bevacizumab and chemotherapy in patients with EGFR- or ALK-mutated non-small-cell lung Cancer (ATTLAS, KCSG-LU19-04). J Clin Oncol. (2024) 42:1241–51. doi: 10.1200/JCO.23.01891, 37861993 PMC11095857

[52] Chen Y Du C Shen S Zhang W Shan Y Lyu A . Toripalimab plus bevacizumab as first-line treatment for advanced hepatocellular carcinoma: A prospective, multicenter, single-arm. Phase II Trial Clin Cancer Res. (2024) 30:2937–44. doi: 10.1158/1078-0432.CCR-24-0006, 38687583

[53] Cartoni C Breccia M Giesinger JM Baldacci E Carmosino I Annechini G . Early palliative home care versus Hospital Care for Patients with hematologic malignancies: A cost-effectiveness study. J Palliat Med. (2021) 24:887–93. doi: 10.1089/jpm.2020.0396, 33270529

[54] Li BX Wang YQ Yi YY Zhou N Lv ZX Ma R . The usage and costs of national drug price-negotiated anticancer medicines in a first-tier city in Northeast China: a study based on health insurance data. BMC Public Health. (2024) 24:1309. doi: 10.1186/s12889-024-18820-3, 38745323 PMC11092061

[55] Yi H Cai M Wei X Cao Y Kuai L Xu D . Evaluation of changes in price, volume and expenditure of PD-1 drugs following the government reimbursement negotiation in China: a multiple-treatment period interrupted time series analysis. J Glob Health. (2025) 15:4069. doi: 10.7189/jogh.15.04069, 40247711 PMC12006830

[56] Ding Y Zheng C Wei X Zhang Q Sun Q. The impacts of the National Medication Price-Negotiated Policy on the financial burden of cancer patients in Shandong province, China: an interrupted time series analysis. BMC Public Health. (2022) 22:2363. doi: 10.1186/s12889-022-14525-7, 36527037 PMC9756446

[57] Zhu Z Zhang J Xu Z Wang Q Qi Y Yang L. Impacts of national reimbursement drug price negotiation on drug accessibility, utilization, and cost in China: a systematic review. Int J Equity Health. (2025) 24:36. doi: 10.1186/s12939-025-02390-w, 39905408 PMC11796270

[58] Shang JA-O Zhou L Huang L Yang F Liu Y Zhang C . Trends in antineoplastic drug use, cost and prescribing patterns among patients with lung cancer in nine major cities of China, 2016-2020: a retrospective observational study based on inpatient and outpatient hospital data (2023) 13:e069645. doi: 10.1136/bmjopen-2022-069645, 36931677 PMC10030656

[59] Xia M Wen J Liu Q Zheng Z Ran Q. Promoting access to innovative anticancer medicines: a review of drug price and national reimbursement negotiation in China. Inquiry. (2023) 60:729. doi: 10.1177/00469580231170729, 37171066 PMC10184198

[60] Zhu H Zhu J Zhou Y Shan L Li C Cui Y . Impact of the national reimbursement drug list negotiation policy on accessibility of anticancer drugs in China: an interrupted time series study (2296-2565 (Electronic)). Front Public Health. 10:1093. doi: 10.3389/fpubh.2022.921093, 35844892 PMC9283976

[61] Rand LZ Kesselheim AS. An international review of health technology assessment approaches to prescription drugs and their ethical principles. J Law Med Ethics. (2020) 48:583–94. doi: 10.1177/1073110520958885, 33021189

[62] Binder L Ghadban M Sit C Barnard K. Health technology assessment process for oncology drugs: impact of CADTH changes on public payer reimbursement recommendations. Curr Oncol. (2022) 29:1514–26. doi: 10.3390/curroncol29030127, 35323327 PMC8947453

[63] Zhou J Lan T Lu H Pan J. Price negotiation and pricing of anticancer drugs in China: an observational study. PLoS Med. (2024) 21:e1004332. doi: 10.1371/journal.pmed.1004332, 38166148 PMC10793910

[64] Gibson L Zimmerman F. Measuring the sensitivity of difference-in-difference estimates to the parallel trends assumption. Res Methods Med Health Sci. (2021) 2:148–56. doi: 10.1177/26320843211061306

